# Influenza surveillance in the Pacific Island countries and territories during the 2009 pandemic: an observational study

**DOI:** 10.1186/1471-2334-13-6

**Published:** 2013-01-07

**Authors:** Jacobus Leen Kool, Boris Igor Pavlin, Jennie Musto, Akanisi Dawainavesi

**Affiliations:** 1 Division of Pacific Technical Support, World Health Organization, Suva, Fiji; 2 Office of the WHO Representative in Papua New Guinea, World Health Organization, Port Moresby, Papua New Guinea

**Keywords:** Influenza, Pacific, Pandemic, H1N1, Epidemiology, Surveillance

## Abstract

**Background:**

Historically, Pacific island countries and territories (PICTs) have been more severely affected by influenza pandemics than any other part of the world. We herein describe the emergence and epidemiologic characteristics of pandemic influenza H1N1 in PICTs from 2009 to 2010.

**Methods:**

The World Health Organization gathered reports of influenza-like-illness and laboratory-confirmed pandemic H1N1 cases from all 23 Pacific island countries and territories, from April 2009 through August 2010. Data were gathered through weekly email reports from Pacific island countries and territories and through email or telephone follow-up.

**Results:**

Pacific island countries and territories started detecting pandemic H1N1 cases in June 2009, firstly in French Polynesia, with the last new detection occurring in August 2009 in Tuvalu. Nineteen Pacific island countries and territories reported 1,972 confirmed cases, peaking in August 2009. No confirmed pandemic H1N1 cases were identified in Niue, Pitcairn and Tokelau; the latter instituted strict maritime quarantine. Influenza-like-illness surveillance showed trends similar to surveillance of confirmed cases.

Seven Pacific island countries and territories reported 21 deaths of confirmed pandemic H1N1. Case-patients died of acute respiratory distress syndrome or multi-organ failure, or both. The most reported pre-existing conditions were obesity, lung disease, heart disease, and pregnancy.

Pacific island countries and territories instituted a variety of mitigation measures, including arrival health screening. Multiple partners facilitated influenza preparedness planning and outbreak response.

**Conclusions:**

Pandemic influenza spread rapidly throughout the Pacific despite enormous distances and relative isolation. Tokelau and Pitcairn may be the only jurisdictions to have remained pandemic-free. Despite being well-prepared, Pacific island countries and territories experienced significant morbidity and mortality, consistent with other indigenous and low-resource settings.

For the first time, regional influenza-like-illness surveillance was conducted in the Pacific, allowing health authorities to monitor the pandemic’s spread and severity in real-time.

Future regional outbreak responses will likely benefit from the lessons learned during this outbreak.

## Background

Historically, Pacific island countries and territories (PICTs) have been more severely affected by influenza pandemics than any other part of the world. As an extreme example, during the 1918 influenza pandemic, Western Samoa (now Samoa) experienced the loss of 19-22% of its population [1]. On the other hand, also during the 1918 pandemic, four Pacific island nations were able to delay or prevent introduction of the influenza virus through strict application of maritime quarantine. Eventually, all experienced infection after the quarantine was lifted, but delay in introduction seems to have helped reduce the impact [1]. One area, the remote Lau and Yasawa islands of Fiji, is the only known Pacific area that appears to have avoided the 1918 pandemic altogether [2]. By 2009, all PICTs except for Tokelau and Pitcairn Islands could be reached by commercial air line, making timely quarantine unrealistic for most.

On 25 April 2009, the World Health Organization (WHO) declared the emergence of novel H1N1 influenza in Mexico a Public Health Emergency of International Concern [3]. In response, PICTs that were not already conducting surveillance were requested to initiate influenza-like illness (ILI) surveillance in their main health facilities.

In the Pacific, the 22 PICTs, consisting of American Samoa, Cook Islands, Federated States of Micronesia, Fiji, French Polynesia, Guam, Kiribati, Marshall Islands, Nauru, New Caledonia, Niue, Commonwealth of the Northern Mariana Islands (CNMI), New Zealand, Palau, Pitcairn Islands, Samoa, Solomon Islands, Tokelau, Tonga, Tuvalu, Vanuatu, and Wallis & Futuna are supported by WHO Division of Pacific Technical Support (WHO DPS) based in Suva, Fiji. For the purposes of this analysis, New Zealand is not included amongst the PICTs. Papua New Guinea (PNG) is included in this report. Thus this paper is based on data received from 23 PICTs.

The objectives of this study were to describe the epidemiology, characteristics (including a summary of fatal cases), and control measures against the H1N1 pandemic in the Pacific, until the declaration of the end of the pandemic on August 10^th^, 2010.

## Methods

During the beginning of the pandemic in May 2009, the WHO DPS requested PICTs to initiate influenza-like-illness (ILI) surveillance (if not already in place) and report cases of ILI and cases of laboratory-confirmed pH1N1. ILI was defined as a case-patient with fever (measured or reported) with cough and/or sore throat. Most PICTs conducted ILI surveillance in one or two locations, usually their main health centre(s).

When cases of ILI were identified, WHO DPS and the Secretariat of the Pacific Community (SPC) requested PICTs to collect two nasopharyngeal samples per patient. Samples were referred to reference laboratories on viral transport media with dry ice or in ethanol, depending on the duration of transport. The majority of PICTs in the South Pacific sent samples to the WHO Collaborating Centre in Melbourne, with the exception of Cook Islands and Samoa, which sent specimens to New Zealand. Countries in the North Pacific referred specimens to Hawaii. Testing was no longer recommended once cases of pH1N1 were confirmed within a country unless PCR was available in-country, as is the case in French Polynesia, Fiji, Guam, and New Caledonia.

WHO Headquarters requested that all Member States report deaths related to laboratory-confirmed pH1N1 using a standardised case summary form; in the Pacific, these were collated by the WHO DPS.

Data were gathered through weekly email reports from the PICTs. Where data were not provided or additional information was needed, email or telephone follow-up was conducted. A weekly summary report was disseminated by WHO through PacNet, the email mailing list and communication tool of the Pacific Public Health Surveillance Network, to which health officials from all PICTs subscribe.

Case counts of ILI and confirmed pH1N1 were tabulated and analysed in Excel 2007 (Microsoft Corporation, Redding, Washington, USA). Maps displaying the number of pandemic influenza H1N1 confirmed cases reported to WHO by PICTs were also created every week by SPC using a prototype Population Geographic Information System called PopGIS [4].

Control measures employed by PICTs were determined through reports from PICTs to WHO DPS and SPC.

## Results

### Epidemiology of ILI and confirmed pH1N1 in PICTs

**Figure 1:**
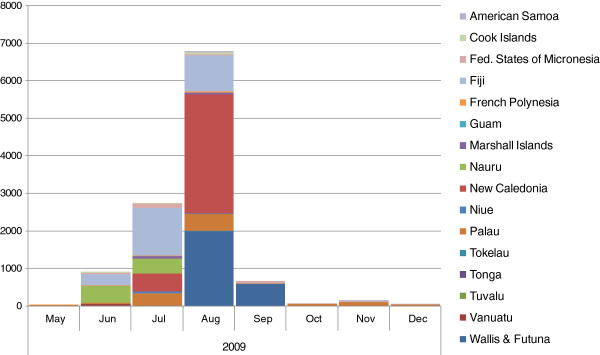
**Reported cases of influenza-like illness, Pacific island countries and territories, June to December 2009. **Countries and territories with no data are not included. Sources: reports to WHO; source for New Caledonia: Inform’Action December 2009.

Hawaii and New Zealand were the first jurisdictions in the Pacific region to detect pH1N1 cases in late April, 2009. The PICTs started detecting ILI cases in May, with cases first being reported in French Polynesia and Palau in May, followed by the rest of the PICTs and finally Tuvalu in August. Sixteen PICTs reported a total of 16,784 cases of ILI, with a peak occurring in August 2009 [Figure 1]. CNMI, PNG, Samoa and Tuvalu did not report ILI data to WHO DPS in 2009.

**Figure 2:**
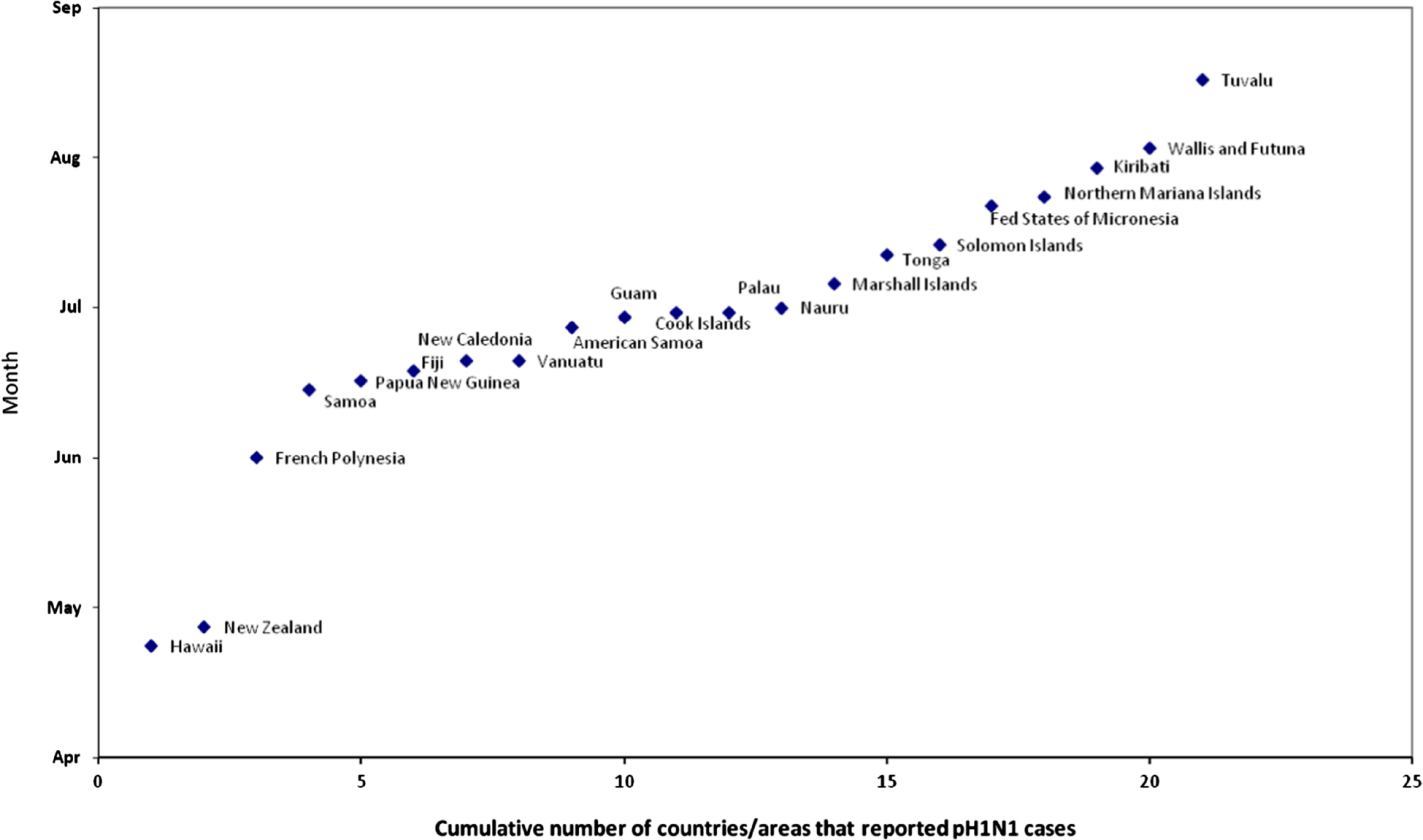
**Timeline of Pacific island countries and territories’ initial reports of human cases of influenza pH1N1. **Date of estimated onset or date of specimen collection of initial lab-confirmed human case is on the vertical axis; cumulative number of PICTs reporting pH1N1 cases is on the horizontal axis.

Confirmed cases of pH1N1 followed the same pattern and were firstly reported from French Polynesia in June, following the arrival of a tourist from Boston, USA, with the last new detection occurring in August in Tuvalu [Figure 2]. Nineteen PICTs reported 1,972 confirmed cases with a peak in week 3 in August 2009. Four PICTs accounted for 64% (n=1,262) of these confirmed cases: New Caledonia (n=501), Guam (n=341), Fiji (n=235), and French Polynesia (n=185). These were the only PICTs that had laboratory capacity to confirm pH1N1 in-country using PCR; other PICTS had to ship their samples for confirmation to an overseas laboratory. Maps displaying the number of pandemic influenza H1N1 confirmed cases reported to WHO by PICTs were also created every week using PopGIS.

### Absence of confirmed pH1N1 in three PICTs

**Figure 3:**
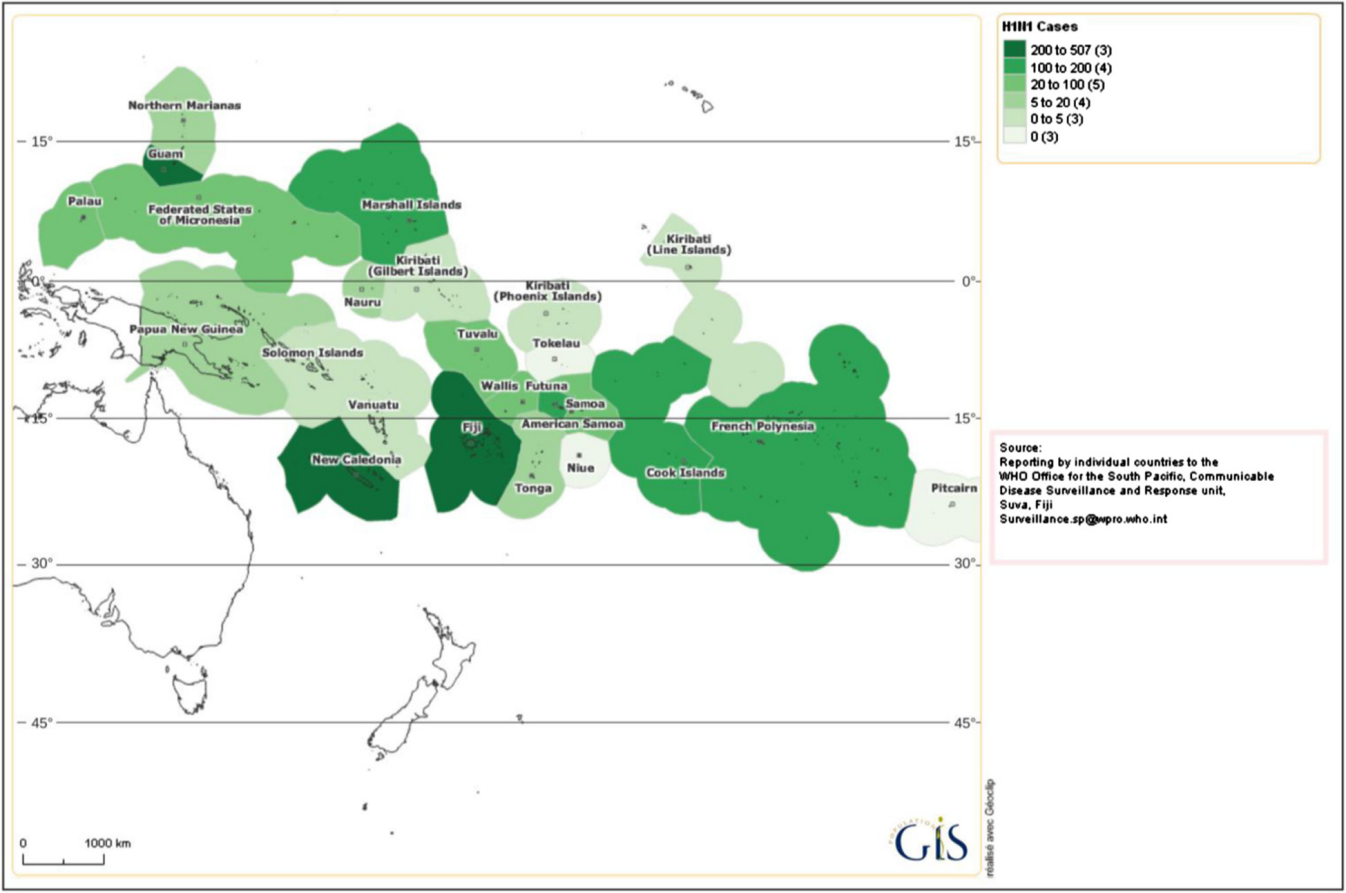
**Map of pandemic H1N1 influenza cases reported to WHO by Pacific island countries and territories, 2009–2010. **Darker shading indicates a higher number of case-patients. Map produced with the online mapping tool SPC PopGIS [6].

No confirmed pH1N1 cases were identified in Niue, Pitcairn and Tokelau [Figure 3].

Niue, a small single-island country with a population of approximately 1,500, can be reached by a weekly flight from Auckland, New Zealand, or by cargo ship, which sails once every 3–4 weeks. The Niue health authorities instituted health screening at the airport and seaport beginning in early May, and coordinated with authorities in New Zealand to screen passengers bound for Niue in Auckland; this resulted in denial of travel for at least 2 symptomatic individuals. A peak of influenza-like-illness occurred in July (61 reported cases). Five nasopharyngeal swabs were sent to New Zealand for influenza testing; all were negative [5]. However, all of these samples were collected late in the outbreak, after fever had subsided in most patients (personal communication, Mr Manila Nosa, Niue health department).

Pitcairn Islands, a British overseas territory with a population of approximately 50, is accessible only by ship from Mangareva, Gambier Islands, French Polynesia, which, in turn, is accessed by a flight from the French Polynesian capital of Papeete [6]. In addition, the main island is a popular stop for cruise ships. Throughout the pandemic, the medical doctor on Pitcairn did not report cases of influenza-like illness among islanders.

Tokelau, a country consisting of 3 atolls with a total population of approximately 1,200 persons, can only be reached by ship, which makes the voyage every 2 weeks from Apia, Samoa; it takes 2 to 4 days to reach each of the atolls. Starting in May 2009, the Tokelau government required that persons planning to travel to one of the atolls wait a minimum of 7 days in Apia before boarding. Persons with ILI symptoms were not allowed to embark. Passengers on the boat were monitored for signs of influenza. Upon disembarkation, passengers were placed in isolation, either on an uninhabited islet, which had been prepared to cater for them, or in dedicated isolation buildings. After another 7 days without symptoms, these persons were finally allowed to join the rest of the population on the atoll. Nine ILI cases were reported; one in June and 8 in August; 5 nasopharyngeal swab samples from these persons were negative for influenza in a reference lab. When the pH1N1 vaccine became available in 2010, Tokelau, with assistance from New Zealand, vaccinated the entire population aged 2 and older [7].

### Deaths

Seven PICTs reported 21 deaths in confirmed case-patients of pH1N1, all of them in 2009 [Table 1]. Three countries completed WHO case summary forms; the remaining case data were elicited by email or telephone.

**Table 1:** Fatal cases reported by country, population and mortality rate

**Country**	**Population**	**Number of fatal cases**	**Deaths per 100,000 population**
Cook Islands	11,000	1	9.1
Guam	182,000	2	1.1
French Polynesia	274,000	7	2.6
Marshall Islands	55,000	1	1.8
New Caledonia	249,000	7	2.8
Samoa	184,000	2	1.1
Tonga	105,000	1	0.9

Sixty-two per cent (n=13) of reported deaths were among females. Ages ranged from 6 weeks to 73 years, with a median age of 27 years.

Among the fatal cases, cough was the most commonly reported symptom (n=18, 86%). Other symptoms reported were fever (n=17, 81%), shortness of breath (n=12, 57%), muscle pain (n=4, 19%), headache (n=4, 19%), vomiting (n=4, 19%), runny nose (n=2, 10%), diarrhoea (n=1), sneezing (n=1) and sore throat (n=1).

Fatal case-patients had an onset of illness from 1 July 2009 to 14 September 2009. Duration of illness prior to death ranged from 1 day to 27 days, with a median of 11 days. Sixty-two per cent (n=13) of case-patients were hospitalised prior to death.

Cause of death was reported for 17 (81%) case-patients. Case-patients died of either acute respiratory distress syndrome (n=7, 33%) or multi-organ failure (n=6, 29%), or a combination of both (n=4, 19%) [Table 2].

**Table 2:** Details of fatal pH1N1 cases in PICTS, 2009

**Age**	**Sex**	**Date of onset**	**Date of death**	**Pre-exisiting condition**	**Antivirals**	**Date of oseltamivir**	**Cause of death**
26 yr	F	01-Jul-09	11-Jul-09	Heart disease	N		ARDS*, MOF**
26 yr	F	04-Jul-09	19-Jul-09	Pregnancy	N		ARDS
36 yr	F	19-Jul-09	24-Jul-09	Lung disease	N		ARDS
22 yr	F	23-Jul-09	06-Aug-09	Pregnancy	Y	29-Jul-09	MOF
31 yr	F	23-Jul-09	06-Aug-09	Unknown	N		N/A
32 yr	F	01-Aug-09	20-Aug-09	Lung and heart disease, morbid obesity***	Y	N/A	ARDS, renal failure
24 yr	F	03-Aug-09	12-Aug-09	Immunosuppression	N	N/A	N/A
24 yr	F	08-Aug-09	04-Sep-09	Pregnancy	Y	N/A	ARDS, MOF
58 yr	F	09-Aug-09	19-Aug-09	Diabetes, obesity	N		MOF
27 yr	M	10-Aug-09	20-Aug-09	Morbid obesity	Y	14-Aug-09	MOF
30 yr	M	10-Aug-09	21-Aug-09	Nil known	N		ARDS, MOF
46 yr	F	10-Aug-09	22-Aug-09	Nil known	N		ARDS, MOF
11 months	M	13-Aug-09	08-Sep-09	Prematurity	Y	19-Aug-09	ARDS
13 yr	F	15-Aug-09	29-Aug-09	Cerebral palsy	Y	16-Aug-09	ARDS
6 weeks	M	16-Aug-09	17-Aug-09	Nil known	N		N/A
17 yr	M	17-Aug-09	25-Aug-09	Lung disease	Y	24-Aug-09	ARDS, pneumonia
36 yr	F	18-Aug-09	30-Aug-09	Morbid obesity	Y	24-Aug-09	MOF
1 yr	F	22-Aug-09	24-Aug-09	Genetic disorder	N		MOF
73 yr	M	25-Aug-09	28-Aug-09	Heart disease	Y	N/A	MOF
61 yr	M	02-Sep-09	03-Sep-09	Lung disease, morbid obesity	N		N/A
45 yr	M	14-Sep-09	20-Sep-09	Nil known	Y	N/A	ARDS

N/A not available.

*ARDS - acute respiratory distress syndrome.

**MOF - multi-organ failure.

***morbid obesity = body mass index (BMI) of 40 or higher.

Information about pre-existing conditions was available for 20 case-patients. Four (19%) case-patients had no reported pre-existing conditions or risk factors for severe disease. Pre-existing conditions reported were: morbid obesity/obesity (n=5, 24%), lung disease (n=4, 19%), heart disease (n=3, 14%), pregnancy (n=3, 14%), diabetes (n=1), immunodeficiency (n=1), cerebral palsy (n=1), prematurity in an infant (n=1) and genetic disorder (mitochondrial disease) (n=1). Three case-patients were reported to have more than one pre-existing condition/risk factor.

Antiviral therapy (oseltamivir) was prescribed for 10 case-patients (48%) (data available for all case-patients). Antibiotics were prescribed for 10 (48%) case-patients (data not known for 9 case-patients). Four case-patients received both antiviral and antibiotic therapy.

### Control methods

Most PICTs activated their pandemic plans at the start of the pandemic. All PICTs communicated public health messages to their public through a variety of media. These included advice to seek healthcare when ill, especially if one had risk factors; to avoid close contact with symptomatic persons; to stay away from work or school when ill; and to observe good hand- and cough/sneeze hygiene. All PICTs had access to antiviral drugs (oseltamivir), though these were mostly used for treatment rather than prevention. To the best of our knowledge, no PICTs instituted internal controls on movement to limit community spread. In most PICTs (as in many other countries around the world), until cases of pH1N1 were confirmed from within their jurisdictions, authorities placed great emphasis on instituting arrival health screening of airline passengers.

Many PICTs had pre-positioned personal protective equipment, antiviral medications, laboratory equipment and other supplies necessary to control the spread of infection. During the pandemic, WHO received vaccine donations sufficient to immunize 10% of the population of 95 selected countries worldwide. In the Pacific, this included 12 countries (Fiji, Cook Islands, Kiribati, Nauru, Niue, Papua New Guinea, Samoa, Solomon Islands, Tokelau, Tonga, Tuvalu, and Vanuatu); there was, however, substantial delay in the receipt of vaccine in several of these jurisdictions and most received vaccine in early 2010. Some PICTs, notably those affiliated with large metropolitan governments, i.e. Cook Islands, Tokelau, Niue, French Polynesia, and New Caledonia, received vaccine late 2009. Situation updates and control advice were provided by regional partners such as WHO, SPC, and the US Centers for Disease Control and Prevention (CDC).

## Discussion

Pandemic influenza spread rapidly throughout the Pacific despite enormous distances and relative isolation. PICTs that first identified pH1N1 were those that receive many tourists, either by cruise ship or plane, from countries where pandemic influenza had already been circulating. Only three jurisdictions did not report confirmed cases. Of these, Niue experienced an outbreak of influenza-like illness during high pandemic influenza activity in the region, so it is not possible to exclude the possibility that pH1N1 infection was present, despite several negative swab samples. Pitcairn’s tiny population of approximately 50 may have experienced a small number of influenza-like-illness cases, which went undetected, or perhaps their extreme isolation prevented introduction of the virus. Tokelau thus may have been the only country in the world that was able to keep the pandemic out, through strict maritime quarantine. To verify these findings, serosurveys could be considered.

Authorities in several PICTs placed great emphasis on instituting arrival health screening of airline passengers; in many cases, this came at significant expense and at the sacrifice of routine public health services because healthcare workers were diverted from their regular duties to staff airport arrival areas. Most of these screening efforts involved measurement of temperature, either with infrared detection devices or with plain thermometers. While it is difficult to draw any firm conclusions, it is likely that this approach was ineffective in substantially delaying arrival of influenza in any PICT, due to the low sensitivity of the temperature screening method [8]; the fact that many passengers would have taken paracetamol or other NSAIDs to reduce a fever; and the possibility of arrival of infected passengers still in the incubation period. WHO and other agencies therefore advised focusing instead on community-level surveillance and control measures [9].

Most PICTs were well-prepared for the arrival of the pandemic, as there had been a strong emphasis prior to the pandemic on pre-pandemic planning and capacity-building by WHO, USCDC, and SPC, the latter through the “Pacific Regional Influenza Pandemic Preparedness Project” (PRIPPP) funded by Australia and New Zealand. One challenge faced by PICTs during the pH1N1 pandemic was the difficulty in applying advice aimed at the global level to the unique local circumstances found in the Pacific. Notably, several PICT authorities experienced frustration at being advised that countries should not institute border closure, while at the same time being cognizant of the history of the 1918 influenza pandemic in the Pacific and the very real possibility of excluding infection by instituting strict border controls. All PICTs had pandemic preparedness plans in place, though, as in other countries, challenges were encountered when trying to apply these plans, developed for worst-case-scenarios along the lines of a pandemic of highly pathogenic avian influenza, to a pandemic with a much milder profile. For instance, several PICTs had plans that called for automatic closure of borders upon the declaration of Pandemic Phase 6. A few PICTs closed their air- and seaports to travelers for up to two weeks but found it impossible to sustain this measure for the long term. A major lesson learned, therefore, was the need to make future pandemic (or emerging disease) plans flexible enough to accommodate varying degrees of severity.

Four PICTs reported 63% of all cases of pH1N1. Each of these PICTs had laboratories able to confirm pH1N1, which likely resulted in a greater proportion of ILI cases being confirmed. Further, laboratory capacity in these PICTs resulted in their cases being reported closer to the true onset date of the case and so it appears that the pandemic arrived earlier in these countries than in countries that relied on overseas laboratories to confirm the diagnosis. Once pH1N1 was identified in a PICT, confirmatory testing in overseas laboratories was no longer considered a priority for those PICTs without laboratories, thus underestimating the number of confirmed cases in these countries.

The 2009 influenza H1N1 pandemic had a relatively low impact in the Pacific, as compared with previous pandemics such as 1918 [1]. Nevertheless, the Pacific experienced significant morbidity and mortality, consis-tent with the experiences of other indigenous and low-resource settings throughout the world [10-12]. The highest mortality rate was in the Cook Islands with 9.1 deaths reported per 100,000 inhabitants. The mortality rates in the PICTs were ten-fold higher than most other countries reported during the same period [13]. The reasons for this are probably multifactorial, including: indigenous people and Pacific islanders have higher rates of pre-existing conditions, such as heart and lung disease, diabetes and obesity when compared with non-indigenous populations; there are relatively large numbers of children and pregnant women; access to health care and diagnostic services is often limited or delayed; and, larger family size and social networks, crowding, and poverty may increase the risk of infection. Our data also show that, during this pandemic, 19% of people who died did not have a pre-existing condition, a younger population was severely affected, and pregnant women were at risk for severe disease. These findings are consistent with other published reports [14-16]. Unfortunately, because most PICTs collected demographics and risk factor data only on fatal cases of pH1N1, it was not possible to perform statistical analysis to determine which, if any, of these risk factors were significantly associated with a fatal outcome.

The computed case fatality rate (CFR) (1%), amongst laboratory-confirmed cases in the Pacific, appears high compared with published rates from other regions. However, these findings must be interpreted with caution. More severe cases of influenza are more likely to be tested and diagnosed with H1N1, which will over-estimate the CFR. Additionally, the majority of cases of influenza are not laboratory-confirmed due to limited laboratory capacity in PICTs, and limited influenza surveillance and detection of clinical case-patients in some places. Therefore, it is likely that the true number of case-patients of pandemic influenza H1N1 is far greater than diagnosed. It is also likely that not all deaths due to pandemic influenza H1N1 were recognised and reported. The mortality data have several limitations. Data collection was not standardised, as only three countries completed WHO case summary forms. The remaining case data were elicited by email or telephone and as a result data are incomplete for many case-patients. Pre-existing conditions are not well defined, so it is possible that different definitions are used within PICTs, e.g. heart disease may or may not include primary hypertension, and obesity may be defined as a body mass index greater than 30, 35 or even 40. Interpretation of the importance of pre-existing conditions such as heart disease and obesity is therefore not possible. Onset dates were estimated by reporting parties where information was not available. Finally, these data relate to a small number of cases.

Syndromic surveillance of influenza-like-illness showed a peak in cases in August and was a good predictor for subsequent laboratory confirmation of pH1N1. As with confirmed cases, the data on ILI should be interpreted with caution as ILI surveillance varied in extent between different PICTs and varied in intensity with the evolution of the pandemic. Further, the method of case ascertainment varied considerably amongst jurisdictions (for example, whether fever had to be documented or could merely be subjective), making it difficult to compare jurisdictions.

## Conclusions

For the first time, regional ILI surveillance was conducted in the Pacific. This allowed PICTs and regional public health organizations to monitor the spread and severity of the pandemic in real-time. Establishing routine syndromic surveillance in PICTs is ongoing but appears to be an acceptable and useful tool for outbreak detection in these jurisdictions [17,18].

Future regional outbreak responses will likely benefit from the experiences and lessons learned during this influenza pandemic.

## Competing interests

The authors declare that they have no competing interests.

## Authors’ contributions

JLK provided overall guidance to surveillance system design and data collection, analyzed data and contributed to the text of the manuscript. BIP, JM, and AD collected and analyzed data and contributed to the text of the manuscript. All authors read and approved the final manuscript.

## Pre-publication history

The pre-publication history for this paper can be accessed here:

http://www.biomedcentral.com/1471-2334/13/6/prepub
